# Predictors of COVID-19 Infection: A Prevalence Study of Hospitalized Patients

**DOI:** 10.1155/2021/6213450

**Published:** 2021-10-13

**Authors:** Huilan Tu, Hong Zhao, Junwei Su, Wenrui Wu, Kaijin Xu, Jianhua Hu, Xuan Zhang, Meifang Yang, Jifang Sheng

**Affiliations:** State Key Laboratory for the Diagnosis and Treatment of Infectious Diseases, Collaborative Innovation Center for the Diagnosis and Treatment of Infectious Diseases, National Clinical Research Center for Infectious Diseases, The First Affiliated Hospital, Zhejiang University School of Medicine, Hangzhou 310003, China

## Abstract

**Aim:**

To find the predictors of coronavirus disease 2019 (COVID-19) in hospitalized patients.

**Methods:**

A prevalence study compared the characteristics of COVID-19 patients with non-COVID-19 patients from January 19, 2020, to February 18, 2020, during the COVID-19 outbreak. Laboratory test results and pulmonary chest imaging of confirmed COVID-19 and non-COVID-19 patients were collected by retrieving medical records in our center.

**Results:**

96 COVID-19 patients and 122 non-COVID-19 patients were enrolled in this study. COVID-19 patients were older (53 vs. 39; *P* < 0.001) and had higher body mass index (BMI) than non-COVID-19 group (24.21 ± 3.51 vs. 23.00 ± 3.27, *P* = 0.011); however, differences in gender were not observed between the two groups. Logistic regression analysis showed that exposure history (OR: 23.34, *P* < 0.001), rhinorrhea (odds radio (OR): 0.12, *P* = 0.006), alanine aminotransferase (ALT) (OR: 1.03, *P* = 0.049), lactate dehydrogenase (LDH) (OR: 1.01, *P* = 0.020), lymphocyte (OR: 0.27, *P* = 0.007), and bilateral involvement on chest CT imaging (OR: 23.01, *P* < 0.001) were independent risk factors for COVID-19. Moreover, bilateral involvement on chest CT imaging (AUC = 0.904, *P* < 0.001) had significantly higher AUC than others in predicting COVID-19.

**Conclusions:**

Exposure history, elevated ALT and LDH, absence of rhinorrhea, lymphopenia, and bilateral involvement on chest CT imaging provide robust evidence for the diagnosis of COVID-19, especially in resource-limited conditions where nucleic acid detection is not readily available.

## 1. Introduction

The 2019 novel coronavirus (2019-nCoV), also known as the severe acute respiratory syndrome coronavirus 2 (SARS-CoV-2), is a newly emerging infectious virus that is responsible for the spread of coronavirus disease 2019 (COVID-19) worldwide [1, 2]. Because of its relatively high transmissibility and morbidity, the World Health Organization has declared that the outbreak of COVID-19 constituted a public health emergency of international concern and should be considered as a pandemic [3]. Notwithstanding severe mobility restrictions established by governments, over 24,000,000 confirmed cases and 800,000 deaths worldwide were reported to the WHO until August 27, 2020. In the meantime, confirmed COVID-19 cases kept increasing.

In the first case series of COVID-19 published on January 24, 2020, it was found that typical SARS-CoV-2 pneumonia shared similar clinical features and radiological manifestations with the severe acute respiratory syndrome (SARS), and a mortality rate of 15% was reported [4]. Meanwhile, laboratory abnormalities, including lymphopenia, prolonged prothrombin time, and elevated lactate dehydrogenase, have been documented in COVID-19 patients [5]. Radiological findings such as focal ground-glass opacities (GGOs) and/or consolidations characteristics have been reported to be common in COVID-19 patients [6]; however, these findings exhibited poor specificity compared to other types of viral pneumonia. Recent studies suggest that about one-fourth of patients with confirmed COVID-19 had no specific exposure history [6]. Due to the highly similar clinical, laboratory, and radiological characteristics, combined with an unclear exposure history, clinical providers can only diagnose COVID-19 based on the results of the real time-polymerase chain reaction (RT-PCR) in upper respiratory specimens, which is currently advocated by the WHO [7, 8]. However, in areas with limited sources, frequent RT-PCR detection is not a feasible, and cost-effective approach.

Our study investigated the differences in epidemiological, demographic, clinical, laboratory, and radiological characteristics between confirmed COVID-19 patients and suspected patients ruled out for COVID-19 after RT-PCR detection at our hospital. Further multivariate analysis was conducted to explore the clinical predictors of COVID-19. We successfully identified predictors of COVID-19 disease and provided new evidence to help in diagnosis of COVID-19, especially in areas with resource-limited conditions where nucleic acid detection is not readily available.

## 2. Methods and Patient Selection

### 2.1. Patient Selection

All patients with confirmed COVID-19 and suspected COVID-19 were admitted to the isolation wards separately. This study enrolled all admitted patients in our center from January 19, 2020, to February 18, 2020. A total of 96 patients were diagnosed with SARS-CoV-2 infection, and 186 suspected patients were excluded from COVID-19 based on negative RT-PCR results after admission. Only 122 suspected patients were enrolled into the non-COVID-19 group since the remaining patients did not undergo chest imaging. Finally, enrolled patients were separated into a COVID-19 group (*n* = 96) with laboratory-confirmed SARS-CoV-2 infection and a non-COVID-19 group (*n* = 122) for suspected patients excluded from COVID-19 after RT-PCR detection. According to the guideline “Diagnosis and Treatment Protocol for Novel Coronavirus Pneumonia (Trial Version 7)” released by the National Health Commission and State Administration of Traditional Chinese Medicine on March 3, 2020 [9], exposure history was defined as “contact with confirmed COVID-19 patients, travel to or be resident in Wuhan or surroundings within 14 days, or clustering occurrence,” and related clinical manifestations were defined as fever and/or respiratory symptoms, typical lesions (GGOs or consolidations) in chest CT image, normal or decreased white blood cell count, and decreased lymphocyte count. Patients with at least three clinical manifestations or at least two clinical manifestations and a history of specific exposure to COVID-19 met the criteria for COVID-19 suspicion.

The study was conducted in compliance with the “Ethical Principles for Medical Research Involving Human Subjects” of the Helsinki Declaration and was approved by the Ethics Committee of the First Affiliated Hospital of Zhejiang University School of Medicine (no. 2020-IIT-39). Participation to our study was voluntary, and all participants signed an informed consent form before entering the study.

### 2.2. SARS-CoV-2 Testing

Nasopharyngeal swab or sputum was tested for viral nucleic acid detection through reverse transcription-polymerase chain reaction (RT-PCR) (Shanghai BioGerm Medical Biotechnology Co., Ltd.). The exclusion criteria for suspected patients included at least two negative RT-PCR tests taken at least 24 hours apart.

### 2.3. Data Collection

Epidemiological, demographic, clinical, laboratory, and radiological data were collected from all the enrolled patients. Radiographic features of chest CT testing were reviewed independently by two experienced radiologists in our hospital, and points of disagreement were reconciled by a discussion. Lesions on chest CT were described as lobar, unilateral, bilateral, GGO, consolidation, and interstitial changes. Data for all patients were collected using SPSS software. An attending doctor reviewed the data obtained from the electronic medical records. Another doctor double-checked the database against the data collected to ensure accuracy and consistency. Epidemiological and clinical presentation data would be confirmed by contacting the patients by phone calls in case of any doubts.

### 2.4. Statistical Analyses

SPSS software for Windows version 26.0 (SPSS Inc., Chicago, IL, USA) was used to analyze the data. Demographic, epidemiological, clinical, laboratory, and radiological data were analyzed by descriptive analyses. Variables were expressed as mean ± standard deviations (SD), median with interquartile, number, or percentage. The differences between groups were assessed with univariate analyses, chi-square, Student's *t*-test, or Mann–Whitney *U* test. The multivariable logistic regression analysis was performed to identify independent predictors of COVID-19. ROC curve analysis was performed to assess diagnostic accuracy and compared by *Z* test (MedCalc Software, Belgium). In all statistical analyses, a *P* value < 0.05 was statistically significant.

## 3. Results

In the non-COVID-19 group (*n* = 122), influenza viruses A and B were detected in four and two patients, respectively. The median age was 48 years (range: 13 to 96 years), and COVID-19 patients (*n* = 96) were significantly older (53 vs. 39; *P* < 0.001) (Table 1). Gender differences were not observed between the two groups; however, patients with COVID-19 had higher body mass index (BMI) than controls (24.21 ± 3.51 vs. 23.00 ± 3.27, *P* = 0.011) (Table 1).

**Figure 1:**
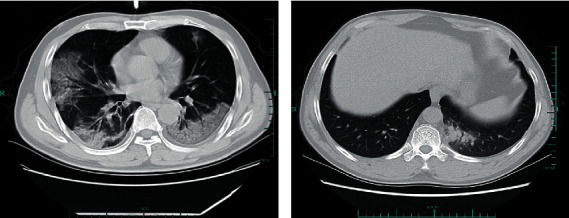
Radiological characteristics of chest CT scan in patients from two groups. (a) Chest CT image showed bilateral ground-glass opacities and consolidations in a 53-year-old male patient with COVID-19. (b) Chest CT image showed focal consolidation with slight ground-glass opacities in the lower lobe of the right lung in a 43-year-old male suspected patient without SARS-CoV-2 infection.

A higher percentage of exposure history was found in the COVID-19 group, compared with the non-COVID-19 group (83.3% vs. 48.4%; odds ratio (OR), 5.34; *P* < 0.001). Patients with COVID-19 were more likely to have comorbidities, including hypertension (39.6% vs. 12.3%; OR, 4.67; *P* < 0.001) and liver disease (14.6% vs. 2.5%; OR, 6.77; *P* = 0.001) (Table 1). In the COVID-19 group, two patients had chronic hepatitis B, and 12 had nonalcoholic fatty liver disease (NAFLD), while three patients in the non-COVID-19 group had NAFLD. There were no significant differences between the two groups for other comorbidities, such as diabetes, coronary heart disease, chronic obstructive pulmonary disease, chronic renal disease, and cancer. Prevalence of symptoms including fever, cough, expectoration, muscle pain, chills, headache, tachypnea, fatigue, abdominal pain, diarrhea, and hemoptysis was comparable in both groups, except for rhinorrhea (28.7% vs. 9.4%; OR, 0.26; *P* < 0.001) and sore throat (21.3% vs. 7.3%; OR, 0.29; *P* = 0.004) which were more common in non-COVID-19 patients. In contrast, patients with COVID-19 had significantly higher hypoxia rates (16.7% vs. 1.7%; OR, 11.8; *P* < 0.001) (Table 1). On admission, chest CT abnormalities were detected in 93 (96.9%) COVID-19 patients and 73 (59.8%) non-COVID-19 patients, among which a significantly greater predominance of bilateral lesions (91.7% vs. 11.5%) and a lower incidence of unilateral lesions (5.2% vs. 48.4%) were found in COVID-19 patients. A greater likelihood of GGOs (53.1% vs. 32.8%; OR, 2.32; *P* = 0.003) and consolidations (72.9% vs. 28.7%; OR, 6.68; *P* < 0.001) on chest CT were found in the COVID-19 group than the non-COVID-19 group (Table 1 and Figure 1). During the comparison of laboratory parameters on admission, COVID-19 patients tended to have lower levels of lymphocyte, monocyte, eosinophil, estimated glomerular filtration rate (eGFR), and serum potassium. Significantly higher levels of alanine aminotransferase (ALT), aspartate aminotransferase (AST), C-reactive protein (CRP), and lactate dehydrogenase (LDH) were found in the COVID-19 group compared to the control group (*P* < 0.05) (Table 2).

**Figure 2:**
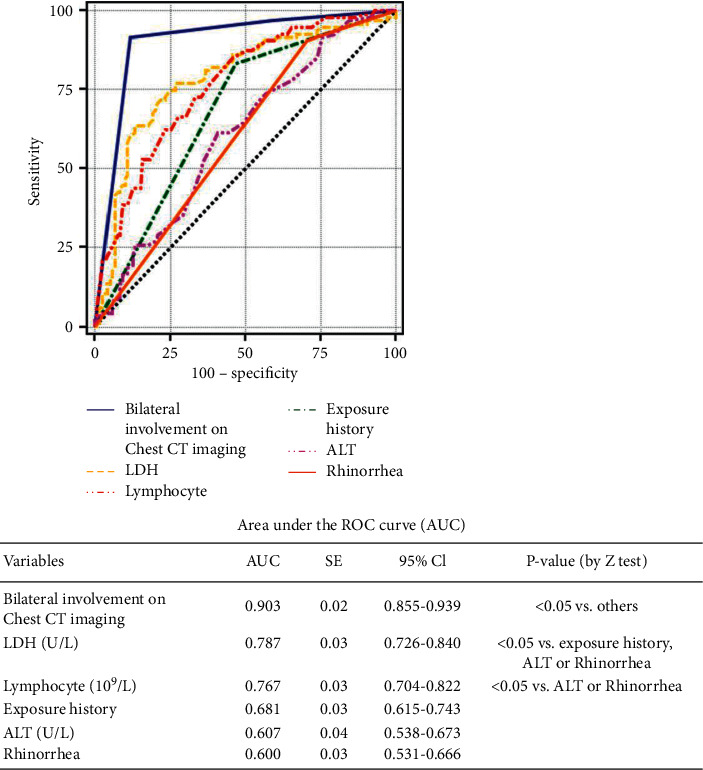
ROC curves models in predicting SARS-CoV-2 infection.

An exploratory multivariate analysis of demographic, epidemiological, clinical, laboratory, and radiological variables associated with SARS-CoV-2 infection was performed. As shown in Table 1, univariate analysis revealed factors associated with the incidence of SARS-CoV-2 infection. Subsequently, variables including age, BMI, exposure history, comorbidities such as hypertension and liver disease, rhinorrhea, sore throat, hypoxia, laboratory values of lymphocyte, monocyte, eosinophil, ALT, AST, eGFR, serum potassium, CRP, LDH, and pneumonia findings on chest CT imaging (GGO and consolidations) were included for binary logistic regression analysis. As shown in Table 3, exposure history (OR: 23.34, 95% CI: 5.63–96.79, *P* < 0.001), runny nose (OR: 0.12, 95% CI: 0.03–0.54, *P* = 0.006), lymphocyte (OR: 0.27, 95% CI: 0.11–0.70, *P* = 0.007), ALT (OR: 1.03, 95% CI: 1.000–1.05,*P* = 0.049), LDH (OR: 1.01, 95% CI: 1.001–1.016, *P* = 0.020), and bilateral involvement on chest CT imaging (OR: 23.01, 95% CI: 8.42–62.88, *P* < 0.001) were independent predictors associated with SARS-CoV-2 infection. Among these six predictors, bilateral involvement on chest CT imaging (AUC = 0.903, *P* < 0.05) had significantly higher predictive value (AUC = 0.903, *P* < 0.05). Similar predictive values were obtained for LDH and lymphocyte count (AUC = 0.767), which were higher than for exposure history (AUC = 0.681), ALT (AUC = 0.607), and rhinorrhea (AUC = 0.600) (Figure 2).

## 4. Discussion

Several studies have sought to leverage differences in the inflammatory process and chest CT imaging to differentiate between COVID-19 and non-COVID-19 patients [10, 11]. However, only a simple comparison of the clinical characteristics of COVID-19 and non-COVID-19 cases was conducted in the study by Zhou et al., and no attempt was made to analyze which features might be inclined to help in COVID-19 diagnosis with negative PCR results [10]. Indeed, it is widely acknowledged that RT-PCR intended for the qualitative detection of nucleic acid from SARS-CoV-2 is currently the most sensitive technique and a prerequisite for confirmed diagnosis [9]. However, the Taiwan Center for Disease Control reported that the positive predictive rate of RT-PCR was only 37.5% for probable SARS cases [12]. Additionally, with the global spread of SARS-CoV-2, dwindling resources are available for medical practitioners, and RT-PCR detection kits may not be available in all outpatient or emergency room settings. This study substantiated that exposure history, elevated ALT and LDH, absence of rhinorrhea, lymphopenia, and bilateral involvement in chest CT provide robust evidence for the diagnosis of COVID-19.

No statistically significant difference in gender was found in both groups. Compared with the non-COVID-19 group, COVID-19 patients were older (mean age: 53 vs. 39; *P* < 0.001). This finding suggested that older patients were more likely to be infected with SARS-CoV-2; meanwhile, older patients have been documented to represent a significant portion of severe cases and deaths associated with COVID-19 [4]. In the present study, patients with COVID-19 had a higher prevalence of comorbidities including hypertension and liver diseases (mainly NAFLD) and higher BMI values. Consistently, hypertension has been reported to be the most common comorbidity in COVID-19, while obesity has been associated with increased susceptibility to SARS-CoV-2 infection [1, 13].

Notwithstanding that fever and cough have been reported as predominant COVID-19 symptoms, no significant difference in both symptoms was found in our study. Alternatively, one of the independent predictors associated with SARS-CoV-2 infection in the present study was rhinorrhea. Importantly, the binding affinity of SARS-CoV-2 to angiotensin-converting enzyme 2 (ACE2) is reported to be one of the most important determinants of virus entry into host cells; however, ACE2 has been found to be rarely expressed in the upper respiratory tract [14]. Interestingly, Zhao et al.'s research revealed that type II alveolar cells were responsible for the predominant expression of ACE2 in lungs of COVID-19 patients, with relatively low expression of ACE2 in other lung cells such as type I alveolar cells, bronchial epithelial cells, endothelial cells, fibroblasts, and macrophages [15]. Accordingly, patients with other acute respiratory infections, such as influenza, were more likely to have upper respiratory symptoms. Furthermore, higher ACE2 expression has been documented in male COVID-19 patients than female patients, which partially explained the higher susceptibility of SARS-CoV-2 infection in males [15].

Compared with the non-COVID-19 group, patients with COVID-19 had lower lymphocyte count and eGFR and higher ALT, AST, CRP, and LDH. During multivariate logistic regression analysis, lymphopenia and higher ALT and LDH levels were important predictors for COVID-19. This result was consistent with data from recent studies, which showed that around 80% of COVID-19 patients presented with lymphopenia on admission and severe COVID-19 patients had lower lymphocyte counts than patients with mild disease [6]. Moreover, increased ALT, AST, CRP, and LDH in severe COVID-19 patients than patients with mild disease were demonstrated in another study [5]. The advanced age of patients and high prevalence of hypertension may explain the lower eGFR in the COVID-19 group. In our study, 16.7% of COVID-19 patients had liver comorbidities accounting for higher transaminase levels compared with the non-COVID-19 group, while excluding the influence of SARS-CoV-2 on the liver [1, 16]. The predicting value of LDH for diagnosis and prognosis in different types of pneumonia has been validated. It is thought that elevated LDH may be due to lung and liver injury [17, 18].

Herein, we found a higher percentage of patients with contact history in the COVID-19 group, which could be used to discriminate COVID-19 from other infections. As the seventh member of the Coronaviridae family capable of causing human infection, SARS-CoV-2 can be transmitted via droplets and direct contact with a basic reproduction number of around 2.2 [19]. Given the high infectivity associated with SARS-CoV-2, COVID-19 cases during the early epidemic phase were geographically close to the local wet market in Wuhan. Accordingly, exposure history was considered a critical risk factor for COVID-19 transmission. Furthermore, the earliest case in Shenzhen involved a family of six members, which was documented as one of the first human-to-human transmission cases in China [20]. These results all suggested exposure was of paramount importance in the screening of COVID-19.

The increasing importance of chest CT imaging in COVID-19 has been proposed. Typical features of imaging findings include bilateral peripheral GGOs and consolidations, reported in the early disease stages [21]. Recent research on the relationship between clinical characteristics and CT images of COVID-19 suggested these typical imaging features were helpful for early COVID-19 screening [22]. Consistently, the same radiological characteristics were found in COVID-19 patients in the present study. Importantly, it has been reported that COVID-19 patients could be asymptomatic and abnormal CT findings often preceded the onset of clinical symptoms [23]. In a study by Xie et al., early chest CT abnormalities in patients with negative RT-PCR results demonstrated the diagnostic value of chest CT in COVID-19 [24]. Consistently, we found that some COVID-19 patients developed typical progressive pulmonary lesions on chest CT while their RT-PCR tests for SARS-CoV-2 were persistently negative (unpublished data). Besides, another study substantiated the discriminative role of CT findings between severe/critical cases and ordinary ones with a sensitivity of 80.0% and specificity of 82.8% [25]. We found that the distribution of pneumonia was relatively atypical in non-COVID-19 patients, which suggested that chest CT imaging, which yielded a high predictive ability for COVID-19 (AUC = 0.903) in our study, had huge prospects in the differential diagnosis of pneumonia of unknown origin. However, in the current study, a simplified classification was used to characterize pulmonary lesions. Indeed, a more refined classification of lesions on chest CT imaging should be used in future studies to increase the robustness of our findings.

There were several limitations in our study. Firstly, due to the retrospective nature of the current study, the limited sample size and bias owing to single-center analysis could lead to interference to detect discriminant factors on presentation. Furthermore, patients excluded due to the nonavailability of chest CT imaging results in the non-COVID-19 group could be a source of selection bias. Recently, a growing body of evidence suggests that other factors affecting our lifestyles, such as nutrition and exercise, could predict COVID-19 [26]. In this regard, appropriate lifestyle changes involving nutrition, exercise, sleep, smoking, and alcohol intake may help prevent severe COVID-19 disease [27]. Our study did not focus on this aspect, which might be another study limitation of our research.

In conclusion, multivariate logistic regression analysis in our study demonstrated that exposure history, elevated ALT and LDH, absence of rhinorrhea, lymphopenia, and bilateral involvement in chest CT imaging were predictors for COVID-19. Importantly, bilateral involvement on chest CT imaging yielded the highest diagnostic value. More studies should be conducted on large-scale populations to substantiate our findings and optimize the diagnostic approach for COVID-19, especially in resource-limited conditions.

## Figures and Tables

**Table 1:** Baseline characteristics of patients in the COVID-19 and non-COVID-19 groups.

Variables	Total (*n* = 218)	COVID-19 (*n* = 96)	Non-COVID-19 (*n* = 122)	OR	95% CI	*P* value
*Demographics*						
Age (years)	48 (32–62)	53 (40–62)	39 (31–61)	—	—	<0.001
Sex (male%)	121 (55.5%)	59 (61.5%)	62 (50.8%)	0.65	0.38–1.12	0.117
BMI (kg/m^2^)	23.52 ± 3.42	24.21 ± 3.51	23.00 ± 3.27	—	—	0.011

*Epidemiology*						
Exposure	139 (63.8%)	80 (83.3%)	59 (48.4%)	5.34	2.81–10.16	<0.001

*Comorbidities*						
Hypertension	53 (24.3%)	38 (39.6%)	15 (12.3%)	4.67	2.37–9.20	<0.001
Diabetes	21 (9.6%)	11 (11.5%)	10 (8.2%)	1.45	0.59–3.57	0.418
CHD	12 (5.5%)	6 (6.2%)	6 (4.9%)	1.29	0.40–4.13	0.669
COPD	9 (4.1%)	3 (3.1%)	6 (4.9%)	0.62	0.15–2.56	0.509
Liver diseases^*∗*^	17 (7.8%)	14 (14.6%)	3 (2.5%)	6.77	1.89–24.32	0.001
CKD	7 (3.2%)	2 (2.1%)	5 (4.1%)	0.50	0.09–2.62	0.652
Cancers^#^	6 (2.8%)	2 (2.1%)	4 (3.3%)	0.63	0.11–3.50	0.906

*Symptoms*						
Fever	187 (86.6%)	84 (87.5%)	103 (85.8%)	1.16	0.52–2.55	0.721
Cough	134 (61.5%)	62 (64.6%)	72 (59.0%)	1.27	0.73–2.20	0402
Expectoration	71 (32.6%)	30 (31.2%)	41 (33.6%)	0.90	0.51–1.59	0.712
Muscle pain	40 (18.3%)	20 (20.8%)	20 (16.4%)	1.34	0.68–2.67	0.400
Chill	19 (8.7%)	8 (8.3%)	11 (9.0%)	0.92	0.35–2.38	0.859
Rhinorrhea	44 (20.2%)	9 (9.4%)	35 (28.7%)	0.26	0.12–0.57	<0.001
Sore throat	33 (15.1%)	7 (7.3%)	26 (21.3%)	0.29	0.12–0.70	0.004
Headache	32 (14.7%)	12 (12.5%)	20 (16.4%)	0.73	0.34–1.58	0.420
Tachypnea	38 (17.4%)	22 (22.9%)	16 (13.1%)	1.97	0.97–4.00	0.058
Fatigue	38 (17.4%)	15 (15.6%)	23 (18.9%)	0.80	0.39–1.63	0.533
Abdominal pain	5 (2.3%)	1 (1.0%)	4 (3.3%)	0.31	0.03–2.82	0.273
Diarrhea	12 (5.5%)	4 (4.2%)	8 (6.6%)	0.62	0.18–2.12	0.442
Hemoptysis	3 (1.4%)	1 (1.0%)	2 (1.6%)	0.63	0.56–7.07	>0.050
Hypoxia ^*ξ*^	18 (8.3%)	16 (16.7%)	2 (1.7%)	11.8	2.64–52.73	<0.001

*Chest CT image*						
GGOs	91 (41.7%)	51 (53.1%)	40 (32.8%)	2.32	1.34–4.03	0.003
Consolidations	105 (48.2%)	70 (72.9%)	35 (28.7%)	6.68	3.68–12.16	<0.001

*Involvement of pneumonia*				—	—	<0.001
Without	52 (23.9%)	3 (3.1%)	49 (40.2%)			
Unilateral	64 (29.4%)	5 (5.2%)	59 (48.4%)			
Bilateral	102 (46.8%)	88 (91.7%)	14 (11.5%)			

^*∗*^Liver disease included chronic hepatitis B and nonalcoholic fatty liver disease. ^#^Cancers involved the lung (*n* = 1), breast (*n* = 1), and colon (*n* = 1) in the COVID-19 group while for the non-COVID-19 group, lung (*n* = 3) and liver cancer (*n* = 1) were present. ^*ξ*^Oxygen saturation < 95%. Data are expressed as mean ± standard deviation, median (Q1-Q3), or number (percent). The group comparison was performed by Student's *t*-test, Mann–Whitney's *U* test, or chi-square test. ALT: alanine aminotransferase; AST: aspartate aminotransferase; BMI: body mass index; CHD: coronary heart disease; CI: confidence interval; CK: creatine kinase; CKD: chronic kidney disease; COPD: chronic obstructive pulmonary disease; CRP: C-reactive protein; CT: computed tomography; diabetes: diabetes mellitus requiring treatment; GGO: ground-glass opacity; LDH: lactate dehydrogenase; OR : odds ratio.

**Table 2:** Comparison of laboratory parameters on admission between the COVID-19 and non-COVID-19 groups.

Variables	Total (*n* = 218)		COVID-19 (*n* = 96)	Non-COVID-19 (*n* = 122)	*P* value
Leukocyte count (10^9^/L)		6.1 (4.5–8.9)	5.7 (4.1–10.1)	6.3 (5.0–8.6)	0.756
Neutrophil (10^9^/L)		4.1 (2.8–6.9)	4.4 (2.8–8.4)	3.9 (2.8–5.8)	0.068
Lymphocyte (10^9^/L)		1.1 (0.7–1.6)	0.8 (0.5–1.2)	1.4 (1.0–1.9)	<0.001
Monocyte (10^9^/L)		0.47 (0.30–0.66)	0.34 (0.23–0.54)	0.51 (0.38–0.76)	<0.001
Eosinophil (10^9^/L)		0.01 (0.00–0.07)	0.00 (0.00–0.01)	0.04 (0.01–0.10)	<0.001
Hemoglobin (g/L)		135.57 ± 24.39	131.97 ± 21.04	138.39 ± 26.48	0.054
Platelet count (10^9^/L)		203 (163–256)	193 (156–243)	215 (172–266)	0.066
Serum bilirubin (*µ*mol/l)		10.5 (6.8–13.9)	11.1 (7.9–15.9)	10.0 (6.2–13.5)	0.061
AST (U/L)		20 (16–30)	22 (17–36)	18 (15–25)	0.003
ALT (U/L)		19 (13–28)	21 (14–37)	16 (12–27)	0.007
Creatinine (*μ*mol/L)		72 (58–84)	72 (60–88)	70 (57–83)	0.244
eGFR (ml/min)		97 (84–112)	94 (74–107)	100 (87–114)	0.008
Serum potassium (mmol/L)		4.0 (3.7–4.4)	3.94 (3.60–4.27)	4.10 (3.81–4.37)	0.011
CK (U/L)		69 (43–101)	66 (40–110)	72 (48–99)	0.787
LDH (U/L)		205 (163–277)	260 (211–340)	176 (152–212)	<0.001
INR		0.98 (0.94–1.03)	0.98 (0.94–1.03)	0.98 (0.93–1.04)	0.884
CRP (mg/L)		14.8 (2.5–44.1)	21.3 (8.8–51.2)	9.1 (1.0–26.2)	<0.001
Procalcitonin (ng/mL)		0.05 (0.03–0.1)	0.06 (0.03–0.09)	0.05 (0.03–0.11)	0.404

Data are expressed as mean ± standard deviation and median (Q1–Q3). Comparisons between groups were performed by Student's *t*-test and Mann–Whitney's *U* test. ALT: alanine aminotransferase; AST: aspartate aminotransferase; BUN: blood urea; CK: creatine kinase; CRP: C-reactive protein; eGFR: estimated glomerular filtration rate; INR: international normalized ratio; LDH: lactate dehydrogenase.

**Table 3:** Predictors of SARS-CoV-2 infection.

Variables	B	OR	95% CI	*P* value
Bilateral involvement on chest CT imaging	3.14	23.01	8.42–62.88	<0.001
Lymphocyte (10 ^9^/L)	−1.29	0.27	0.11–0.70	0.007
LDH (U/L)	0.01	1.01	1.001–1.016	0.020
Exposure history	3.15	23.34	5.63–96,79	<0.001
Rhinorrhea	−2.11	0.12	0.03–0.54	0.006
ALT (U/L)	0.03	1.03	1.000–1.05	0.049

ALT: alanine aminotransferase; B: beta; CT: computed tomography; CI: confidence interval; LDH, lactate dehydrogenase; OR: odds ratio. Statistical analysis was performed using multivariable logistic regression analysis.

## Data Availability

No data were used to support this study.

## Abbreviations

ACE2: Angiotensin-converting enzyme 2
ALT: Alanine aminotransferase
AST: Aspartate aminotransferase
BMI: Body mass index
BUN: Blood urea
CHD: Coronary heart disease
CK: Creatine kinase
CKD: Chronic kidney disease
CI: Confidence interval
COPD: Chronic obstructive pulmonary disease
COVID-19: Coronavirus disease 2019
CRP: C-reactive protein
CT: Computed tomography
Diabetes: Diabetes mellitus requiring treatment
eGFR: Estimated glomerular filtration rate
GGOs: Ground-glass opacities
LDH: Lactate dehydrogenase
INR: International normalized ratio
NAFLD: Nonalcoholic fatty liver disease
OR: Odds ratio
RT-PCR: Real time-polymerase chain reaction
SARS-CoV-2: severe acute respiratory syndrome coronavirus 2.
